# Pertaining to Insurance

**Published:** 1876-12

**Authors:** 


### *PERTAINING TO INSURANCE.*

It has been the endeavor of certain mischief-makers to connect the New Jersey Mutual Life Insurance Company and its managers, in some manner, with the Continental Life, which failed a few weeks ago. A good many wild assertions have been made, some of them slanderous, and all of them tending to injure President Stedwell's company. So he goes to the bottom of things, and shows how flimsy a foundation all these statements stand upon. No one can read the following card without feeling satisfied that the attacks made on the New Jersey Mutual Life have been largely malicious, and are fully met by President Stedwell :

"During the past week various parties have been busy, in the public journals, by verbal statements and by printed circulars, in attacking the history, the business, and the standing of the New Jersey Mutual Life Insurance Company. As we have always sought to conduct our business with fairness and courtesy toward competitors, we have been reluctant to believe that any respectable company would stoop to the low resource of coarse slander. While these attacks emanated merely from malicious and disappointed agents, we refrained from giving them any public notice ; but we now ascertain that the men whose names were first used were but the tools of jealous rivals. We are therefore constrained by a sense of justice to the interests we represent, to make the following statement :

First. The pecuniary condition of this company is excellent, its assets first-class, and its surplus large.

Second. This company is rapidly increasing its business in a legitimate way, issuing over 5,000 new policies annually.

Third. Any statement that this company is interested in any manner in the Continental Life, or in the stock of the Continental Lite, or is endeavoring to save anything for the stockholders of that company, is false.

Fourth. Any statement that this company has any arrangement with the receiver of the Continental, or has received any favors at his hands, is false.

Fifth. Any statement that this company is taking any advantage of the unfortunate policy-holders of the Continental, or is using toward them coercion, misrepresentation or deception, is false.

Sixth. Any statement that this company is issuing only term policies in exchange for Continental policies, is false.

Seventh. Any statement alleging complicity with the Continental officers in any form, or community of interest between persons connected with the two companies, or that any work is being done for individual profit, is false.

Eighth. We regard the men who have been heretofore insured in the Continental as legitimate subjects for life insurance solicitation. We offer to them, as to all other insurable persons, such terms as may be acceptable to them and not injurious to us. No coercion is used; no deception is practiced. We simply propose to utilize the prospective dividends payable on their Continental policies for the purpose of immediate insurance. We propose this upon terms which we regard as liberal to the policy-holders; but we do it not because we owe any protection to them, but only for the reason that we desire, in common with life companies, to increase the volume of our paying business. We are under no obligation whatever to the policy-holders of the Continental. We treat them as we seek to treat all men, with fairness and justice. This is all there is of fire under this dense cloud of smoke.

Ninth. We expect to increase our business, to increase our assets, to increase our income, to increase our surplus, and in doing this to protect many unfortunate policy-holders. Believing this business to be strictly legitimate, we shall protect ourselves from the malicious slanders of enemies by whatever power the law affords." J. H STEDWELL,

President.

---

Nearly a hundred persons were killed or wounded in the recent stampede in the Chinese theatre, in San Francisco. The story now has a new and terrible significance for Eastern readers. The panic was started by a Chinaman who was frightened out of his wits. Some one had carelessly thrown on the floor a lighted cigar or cigarette, and the matting in the gallery had caught fire. The man who first noticed the flames, instead of stamping them out, sprang to his feet, and in the Chinese tongue yelled "Fire! Fire!" at the top of his voice. The panic-stricken audience sprang to their feet and rushed for the door, giving no heed to the actors on the stage, who were as self-possessed and brave as Miss Claxton and Mr.

Studley at the Brooklyn Theatre. Meanwhile, a man in the gallery had taken off his overcoat and smothered the flames. The theatre was saved, but it was too late to stay the panic.

---

A large number of the policy-holders of the defunct Continental Life Insurance Company, of New York, have wisely changed their policies for those of the New Jersey Mutual Life Insurance Company, of Newark, N. J.

---

Our correspondent, P. R. H., is informed that the company he names, and which induced him to insure by means of "cheap" rates, is in bad repute as a contestant of its death claims. Companies making it a practice to contest its losses may well be avoided by the public, although there are frequently cases wherein companies are entirely justified in resisting payment. The company to which our correspondent refers, however, is one that makes litigation a part of its business.

---

As the time rapidly approaches when our life insurance companies are compelled by law to publish their annual statements, those holding policies in these institutions will do well to watch for the exhibit of the ones in which they are interested. Those concerns which fail to give full publicity to their statements, and those which are very dilatory about doing so, should be avoided.

---

In answer to G. W. S. we would say the Clay Fire and Marine Insurance Company, of Newport, Kentucky, is not considered a valuable institution. It has recently been expelled from doing business in the State of New York, and been obliged to reduce its capital to a very small amount.

---

S. T. H.—The companies you name are all reliable and trustworthy.

---

We have no faith in the so called co-operative life insurance companies. They have no capital, and depend entirely on the "pass around the hat" system in the payment of loss claims. The regularly organized and established life insurance companies are the only institutions able to sell reliable indemnity.

---

It is undoubtedly the duty of every man with a family to insure his life in some well established and reputable life insurance company.

---

All improvident and selfish people refuse to insure their lives, but men who love their wives and children do not require to be urged to do so.

